# Communication training for general practitioners aimed at improving antibiotic prescribing: a controlled before-after study in multicultural Dutch cities

**DOI:** 10.3389/fmed.2024.1279704

**Published:** 2024-01-23

**Authors:** Dominique L. A. Lescure, Özcan Erdem, Daan Nieboer, Natascha Huijser van Reenen, Aimée M. L. Tjon-A-Tsien, Wilbert van Oorschot, Rob Brouwer, Margreet C. Vos, Alike W. van der Velden, Jan Hendrik Richardus, Hélène A. C. M. Voeten

**Affiliations:** ^1^Department of Public Health, Erasmus MC, University Medical Center Rotterdam, Rotterdam, Netherlands; ^2^Department of Infectious Disease Control, Municipal Public Health Service Rotterdam-Rijnmond, Rotterdam, Netherlands; ^3^Department of Research and Business Intelligence, Municipality of Rotterdam, Rotterdam, Netherlands; ^4^Pharos, Dutch Centre of Expertise on Health Disparities, Utrecht, Netherlands; ^5^Health Centre Zuidplein, Rotterdam, Netherlands; ^6^Health Centre Levinas, Pharmacy Ramleh, Rotterdam, Netherlands; ^7^Department of Medical Microbiology and Infectious Diseases, Erasmus MC, University Medical Centre Rotterdam, Rotterdam, Netherlands; ^8^Julius Centre for Health Sciences and Primary Care, University Medical Center Utrecht, Utrecht, Netherlands

**Keywords:** antibiotic prescribing, primary care, before-after study, communication skills, cultural differences, physician-patient relationship, respiratory tract infections, ANCOVA

## Abstract

**Introduction:**

Suboptimal doctor-patient communication drives inappropriate prescribing of antibiotics. We evaluated a communication intervention for general practitioners (GPs) in multicultural Dutch cities to improve antibiotic prescribing for respiratory tract infections (RTI).

**Methods:**

This was a non-randomized controlled before-after study. The study period was pre-intervention November 2019 – April 2020 and post-intervention November 2021 – April 2022. The intervention consisted of a live training (organized between September and November 2021), an E-learning, and patient material on antibiotics and antibiotic resistance in multiple languages. The primary outcome was the absolute number of prescribed antibiotic courses indicated for RTIs per GP; the secondary outcome was all prescribed antibiotics per GP. We compared the post-intervention differences in the mean number of prescribed antibiotics between the intervention (*N* = 25) and the control group (*N* = 110) by using an analysis of covariance (ANCOVA) test, while adjusting for the pre-intervention number of prescribed antibiotics. Additionally, intervention GPs rated the training and their knowledge and skills before the intervention and 3 months thereafter.

**Results:**

There was no statistically significant difference in the mean number of prescribed antibiotics for RTI between the intervention and the control group, nor for mean number of overall prescribed antibiotics. The intervention GPs rated the usefulness of the training for daily practice a 7.3 (on a scale from 1–10) and there was a statistically significant difference between pre- and post-intervention on four out of nine items related to knowledge and skills.

**Discussion:**

There was no change in GPs prescription behavior between the intervention and control group. However, GPs found the intervention useful and showed some improvement on self-rated knowledge and communication skills.

## 1 Introduction

The interaction between general practitioners (GPs) and vulnerable patient groups, like immigrants and patients with a low socioeconomic status (SES) has been shown far from optimal (1, 2). Communication barriers between GPs and immigrant patients are common, because of language barriers and cultural aspects influencing communication (3). Suboptimal communication can lead to diagnostic uncertainty, misinterpretation of patients’ reason to consult, feeling pressured, and subsequently inappropriate antibiotic prescribing (4–6). Inappropriate antibiotic prescribing is a common practice among GPs and can induce antibiotic resistance (7, 8).

GP-patient interaction can be improved through multifaceted communication interventions (9–11) that include training skills and the management of vulnerable population groups (12). The training should focus on acquiring *culturally-sensitive communication skills* (13), such as being culturally aware and checking patients’ language ability (14), and on *effective communication skills* that encompass exploring patients’ expectations (15), provide information in smaller portions (16), and make use of the teach back method (17). Along with learning GPs these communication skills, supportive patient materials are required to give arguments why antibiotics are not always needed and to provide suitable alternatives for symptomatic relief (18). Written patient materials are useful in increasing patients’ knowledge (16) and, when used interactively, they increase the effectiveness of interventions to reduce antibiotic prescribing (19–21).

There are only a few studies that have developed an intervention to appropriate antibiotic prescribing focusing specifically on GPs and their immigrant patients (22). As part of the *Prescription of Antibiotics in pRimary CAre* (PARCA)-project, we developed an intervention that focused on improving antibiotic prescribing behavior of GPs by enhancing their communication with immigrant patients through a live group training, an E-learning, and patient information materials. The intervention was implemented in multicultural Dutch cities and focused specifically on managing respiratory tract infection (RTI), as antibiotics are often prescribed inappropriately in these cases (8). However, as the training could also have influenced the prescribing of other antibiotics, we also focused on the total number of prescribed antibiotics. The aim of this study was to evaluate the PARCA intervention, using a non-randomized controlled before-after study design in multicultural Dutch cities.

## 2 Methods

### 2.1 Study design

The design of the study was a non-randomized controlled before-after study (trial registration ID number NL9450). The intervention group consisted of GPs working in multicultural cities. The control group consisted of GPs who were derived from the national database of the Dutch Foundation for Pharmaceutical Statistics (SFK),^1^ the same database as the one that provided data about antibiotic prescribing of the intervention GPs. The selection of control GPs focused on GPs working in the same cities/deprived areas as the intervention GPs. Because of privacy issues, the SFK selected the control GPs so that they could remain anonymous to us.

### 2.2 Study setting

We included GPs working in the three largest Dutch cities: Rotterdam, Amsterdam, and The Hague. These cities contain the largest proportion of inhabitants with an immigration background (i.e., born abroad or having at least one parent who was born abroad); respectively 52, 56, and 56% (23). We primarily focused on GPs who worked in a deprived area. These areas were defined by the Dutch Healthcare Authority (NZa) by considering the percentage of unemployed, low-income residents, and non-Western or Middle East European immigrants living in that area.

### 2.3 Eligibility criteria

All GPs with an interest in improving their communication with immigrant patients and/or patients with a low health literacy were considered for enrolment in the intervention group. We applied the following inclusion criterion; the use of one’s own individual identification code (in Dutch: AGB-code) to prescribe medication. This allowed the extraction of antibiotic prescribing data from the SFK database. We excluded GPs for whom we could not obtain complete prescription data pre- and post-intervention through their individual identification code. We used a cut-off point of <10 prescribed antibiotics because we assumed that in those cases the individual identification code had not been used consistently. This cut-off point was based on a study presenting antibiotic prescription data (24). The control group consisted of anonymous GPs working in deprived areas of the three cities. Based on the registration data of deprived neighborhoods of the NZa, SFK included all GPs from deprived areas as control group, after filtering out the intervention GPs.

### 2.4 Recruitment

The active recruitment of intervention GPs was between February and September 2021. The primary researcher and a research-assistant approached GP practices directly by phone and contacted managers to offer the training as an in-company training. Furthermore, we used other recruitment methods like professional networks (25). After enrollment, individual mailings were used to remind the intervention GPs about following the E-learning, the date and location of the training, and filling in the questionnaire. Because GPs in the control group remained anonymous to us, we were unable to collect data about their individual characteristics.

### 2.5 Intervention

**Figure 1:**
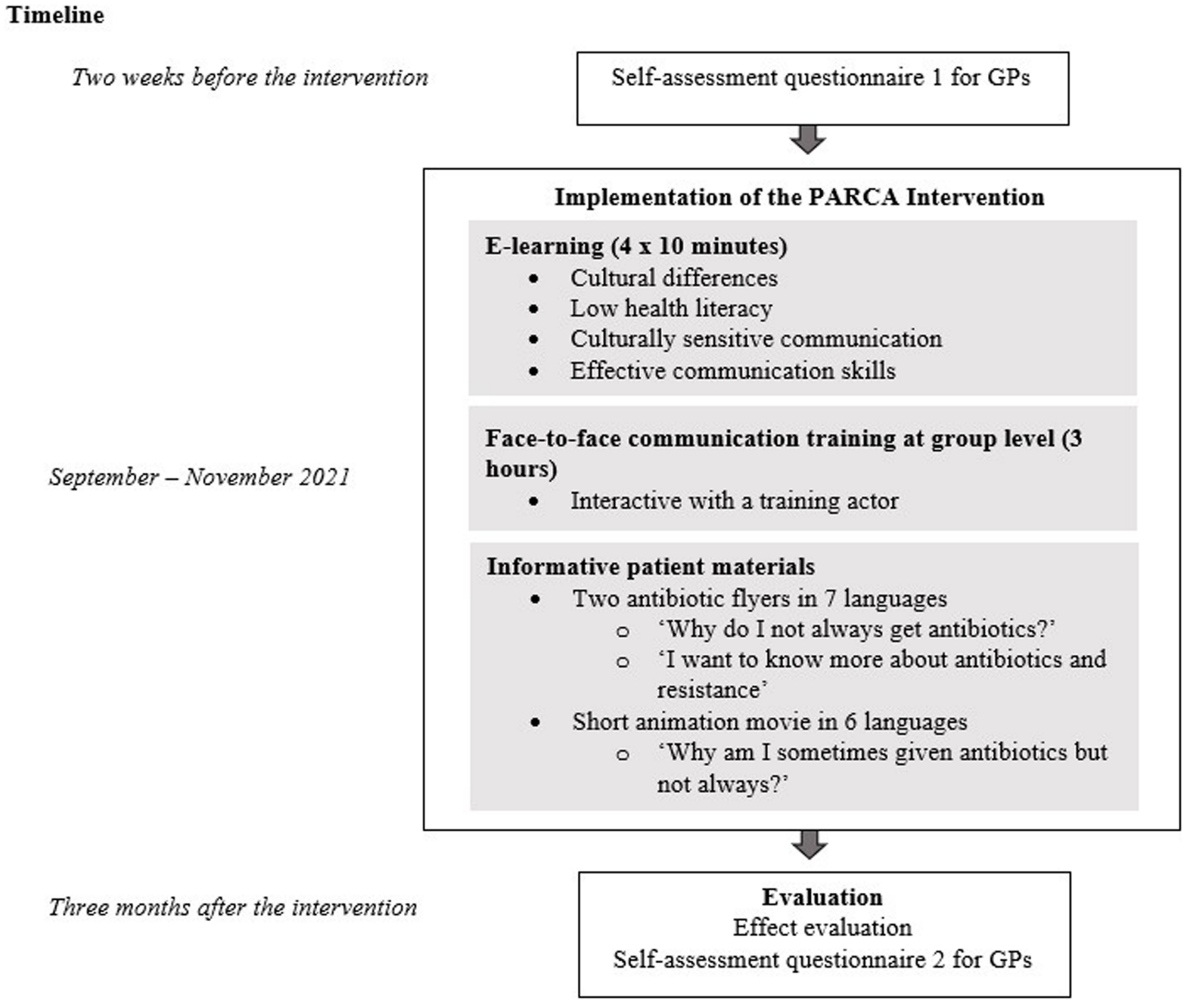
Outline of the PARCA study.

The intervention consisted of three elements (Figure 1). The first element was an E-learning of four modules of 10 min each, all with a focus on antibiotics. The second element was a face-to-face communication training session of three hours at group level, guided by trainers of the national center of expertise on health disparities (Pharos). The third element consisted of simple, informative patient materials, available via the website of the Dutch College of General Practitioners (Thuisarts.nl), that could be used by GPs as support during consultation or in the waiting room. A full description of the intervention elements has been reported elsewhere (25).

### 2.6 Participant timeline and participation

We organized six live training-groups with on average six GPs per training (in total 37 GPs) between September and November 2021. GPs were requested to follow the E-learning modules within 2 weeks before the live training session. During the live training, the GPs received the patient information materials (hard copy and online). Two weeks after the live training, they were reminded by mail about using the online patient materials.

### 2.7 Data collection

For the number of prescribed antibiotics, we used data on the number of dispensed antibiotics as a proxy. These data were obtained from the national database of the SFK. SFK collects dispensing data from 95% of the Dutch community pharmacies. Because these pharmacies are the data owners, data collection is done according to predetermined processing agreements. We compared data post-intervention (November 2021–April 2022) with data pre-intervention (November 2019–April 2020). The data for the pre- and post-intervention period were collected in the winter months to coincide with the seasonal increase in antibiotic prescriptions seen during the winter. As defined per the protocol (25), the pre-intervention period was chosen to be winter ‘19/‘20, because data from winter ‘20/‘21 were too much influenced by COVID-19 (i.e., low antibiotic prescription rates). All data were retrieved retrospectively by SFK in the summer of 2022. Data of the intervention GPs were obtained by their AGB-code and name. Data on the background characteristics of each intervention GP (sex, age, years of work experience, number of FTE, city, and the percentage of patients with a migration background in their practice) were collected through online or hardcopy registration questionnaire before the start of the intervention.

### 2.8 Sample size

Assuming a decrease in the absolute number of prescribed antibiotics for RTI of 16.6% (from 240 to 200 prescriptions per 1,600 patients in 6 months’ time), a standard deviation of 56 per 1,600 patients, and a correlation (Pearson’s *r*) of 0.40 between pre- and post-intervention, the study would require 58 GPs to obtain 80% power at a significance level of 5%; 29 for the intervention group and 29 for the control group.

### 2.9 Outcomes

The primary outcome was the mean number of prescribed antibiotic courses, qualifying for RTI in primary care, per GP. Based on expert opinion and the Dutch antibiotic guidelines, we selected eight first and second choice antibiotics qualifying for RTI in primary care: Doxycycline (J01AA02), Amoxicillin (J01CA04), Amoxicillin/clavulanic acid (J01CR02), Phenoxymethylpenicillin (J01CE02), Pheneticillin (J01CE05), Macrolides (J01FA), Moxifloxacine (J01MA14), and Sulphonamides in combination with trimethoprim (J01EE) (25). The secondary outcome was the mean number of all prescribed antibiotic courses per GP. SFK selected only oral antibiotics and removed chronic-repeat prescriptions for the same antibiotic within two times the duration of the first prescription.

### 2.10 Statistical analysis

The number of prescribed antibiotics for RTI was calculated for each individual GP in the intervention and control group by adding up the total numbers of the selected antibiotics. The number of prescribed antibiotics and the GP characteristics of the intervention GPs were analyzed using descriptive statistics. One-way ANCOVA (analysis of covariance) was used to examine whether there was a difference in the mean number of prescribed antibiotics between the intervention and the control group, while adjusting for the pre-intervention number of prescribed antibiotics (26). Because of a non-normal distribution of the primary and secondary outcomes, we transformed the data by using LOG10 transformation. After the transformation, the assumptions for performing an ANCOVA were met (26). To increase the interpretability of the results, we present back-transformed data in the tables and figures. ANCOVA was performed for per-protocol (PP) analyses, including only intervention GPs who had participated in the intervention, as well as for intention-to-treat (ITT) analysis in which all intervention GPs were included, regardless of their actual participation in the intervention. We analyzed data using the Statistical Package for Social Sciences, version 28.1 for Windows (IBM SPSS Statistics for Windows, Armonk, NY: IBM Corp) and considered 2-sided *p* values less than 0.05 significant.

### 2.11 Self-assessment questionnaire among GPs

All intervention GPs were eligible to participate in the self-assessment questionnaire, also those who were excluded for the primary and secondary outcome analyses. A week before the start of the intervention, they filled out a short online questionnaire to rate their skills in culturally-sensitive communication, assessing patient expectations and explaining antibiotic non-prescribing. Additionally, they rated their own knowledge about different patient groups and communication aspects. The questionnaire contained multiple choice and Likert scale questions (10-point scales). Immediately after the intervention, the GPs rated the usefulness of the training elements for daily practice (10-point scales). Finally, three months later, the GPs received the same self-assessment questionnaire, to measure any change in self-rating and in the perceived usefulness of the training for daily practice. Additionally, they were asked whether they perceived the separate elements of the intervention to be useful and whether the developed patient materials were applicable in daily practice. We also asked about external influences other than the PARCA-intervention that could have affected their antibiotic prescribing behavior. For the evaluation of the statistical significance of changes to GPs’ responses over time, we used the Wilcoxon signed-rank test and considered 2-sided *p* values less than 0.05 significant.

## 3 Ethics

### 3.1 Informed consent

We obtained digital or hard-copy informed consent prior to the start of the study from both the GPs and the pharmacies (the dispensing data owners) in which they agreed to share data about prescribed antibiotics related to individual identification codes of the GPs. For the control group, informed consent was not required because of the processing agreement of the SFK with the affiliated pharmacies, which delineates when anonymous data (i.e., without GP or patient information) about prescribed medications can be used for scientific research.

### 3.2 Confidentiality

Identifying personal information of the intervention GPs was removed and replaced by study numbers. Only the main researcher could access the file containing the key between study numbers and identifying personal information of GPs. SFK only provided aggregated dispensing data per GP without any patient information. Data from separate data files were linked through GPs’ study numbers.

### 3.3 Research ethics approval

Ethical approval for this study was waived by the Medical Ethics Review Committee of the Erasmus MC, University Medical Center Rotterdam (MEC-2020-0142) since the intervention targeted GPs and we did not analyze or include patients’ health outcomes.

## 4 Results

As a result of the inclusion criterion (prescribing antibiotics under one’s own individual identification code), 12 (32.4%) of the 37 intervention GPs and 48 (30.4%) of the 158 control GPs were excluded from data analysis. Characteristics of the remaining intervention GPs (*N* = 25) are presented in the Supplementary Table S1. Most were female (76.0%) and had more than 10 years’ work experience as a GP (44.0%). More than three quarters of the intervention GPs were situated in Rotterdam (76.0%) and served patients from deprived areas (76.0%). The data of the control group consisted of 110 GPs; 46 from Amsterdam, 37 from Rotterdam, and 27 from The Hague.

A new power calculation, based on the randomization ratio of 25:110 demonstrated that it was required to include 20 GPs in the intervention group and 86 GPs in the control group to obtain 80% power at a significance level of 5%. For both groups we reached the minimum number of required GPs and, as such, had sufficient power to perform our analyses.

**Figure 2:**
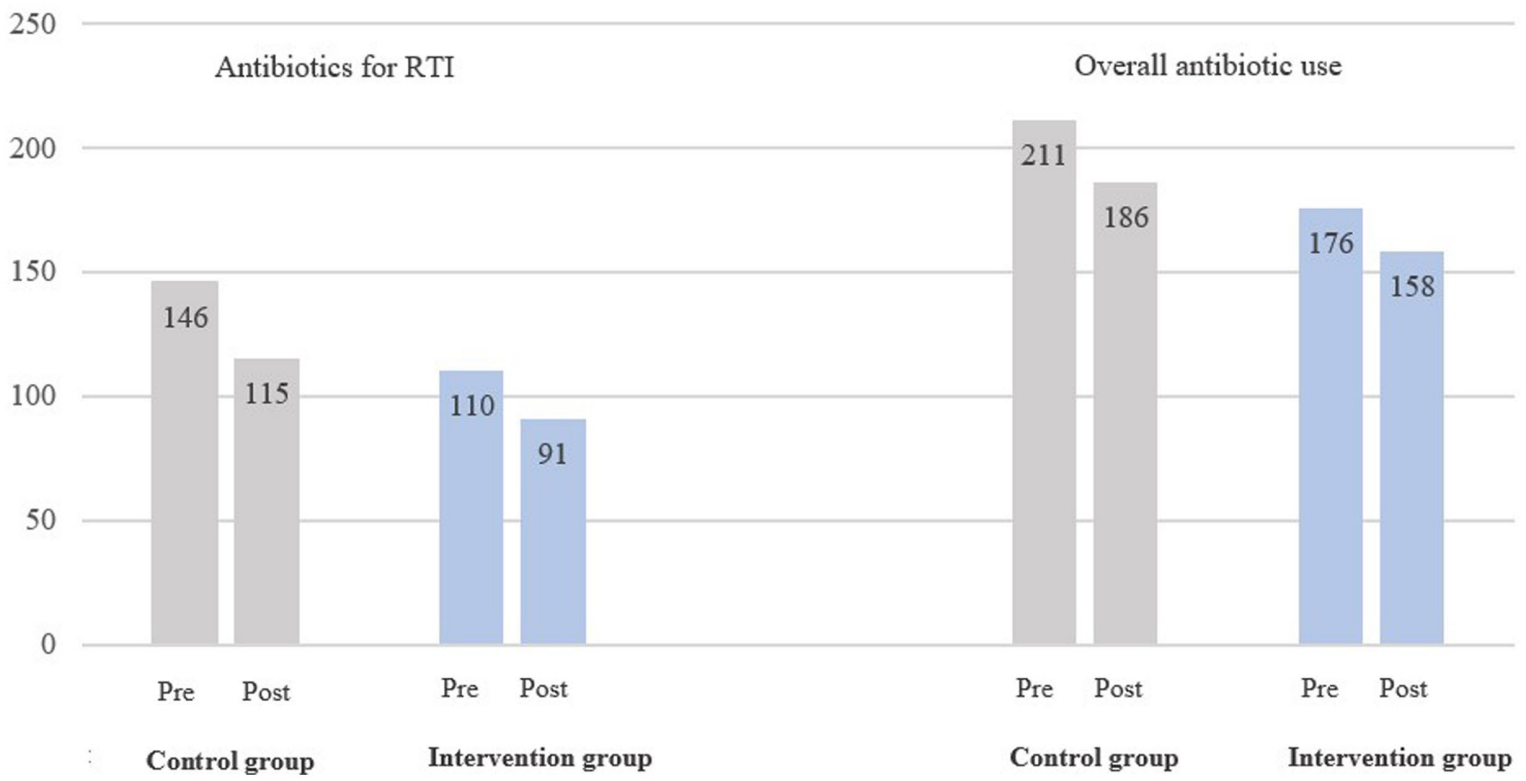
Mean number of prescribed antibiotics per GP, RTI-related and overall, for the intervention group (*N* = 25) and control group (*N* = 110), pre-intervention (2019–2020) and post-intervention (2021–2022). The results were transformed by using LOG10 transformation and back-transformed by using the logarithmic operation in reverse.

The mean number of prescribed antibiotics for RTI decreased from 110 to 91 in the intervention group (−17.3%) and from 146 to 115 in the control group (−21.2%) (Figure 2). The mean number of prescribed antibiotic courses for all infections, decreased from 176 to 158 in the intervention group (−10.2%) and from 211 to 186 in the control group (−11.9%).

There was a statistically non-significant difference of −0.9% (95% CI, −28.2, 37.1%, *p* = 0.96) in the mean number of prescribed antibiotics for RTIs in the intervention group compared to the control group post-intervention, adjusted for the number of prescribed antibiotics pre-intervention (Table 1). For the mean number of all prescribed antibiotics the difference of −4.2% (95% CI, −33.0, 37.1%, *p* = 0.81), was neither significant.

**Table 1:** ANCOVA analysis (intention to treat and per protocol) of the mean number of prescribed antibiotics for RTI per GP and the mean number of all prescribed antibiotics in the intervention group (intention to treat; *N* = 25, per protocol; *N* = 19) compared to the control group (*N* = 110), post-intervention, unadjusted and adjusted for the pre-intervention number of prescribed antibiotics.*

	Post-test			Adjusted post-test		
	Intervention group (per GP)	Control group(per GP)	Difference in the mean number of prescribed antibiotics (intervention vs. control group)	Intervention group (per GP)	Control group (per GP)	Difference in the mean number of prescribed antibiotics (intervention vs. control group)
Intention to treat (ITT)						
Mean number of prescribed AB for RTI	91	115	−20.6%	109	110	−0.9%
95% CI	60–139	94–140	−50.2–26.8%	81–146	96–126	−28.2–37.1%
Value of *p*			0.331			0.960
Mean number of total prescribed AB	158	186	−14.9%	175	182	−4.2%
95% CI	104–240	153–226	−46.2–34.6%	126–241	156–212	−33.0–37.1%
Value of *p*			0.489			0.813
Per protocol (PP)						
Mean number of prescribed AB for RTI	85	115	−26.2%	104	110	−5.6%
95% CI	52–137	94–140	−56.2–24.4%	75–146	96–126	−34.4–35.5%
Value of *p*			0.253			0.751
Mean number of total prescribed AB	145	186	−22.2%	165	182	−9.4%
95% CI	90–233	153–226	−53.7–30.3%	113–240	156–212	−39.6–35.8%
Value of *p*			0.337			0.630

^*^The results were transformed by using LOG10 transformation and back-transformed by using the logarithmic operation in reverse.

Because some GPs did not participate in the intervention (*N* = 6), we excluded them in a per-protocol analysis (PP), which allowed examining the actual effect of the intervention. Comparing the mean number of prescribed antibiotics for RTI, between the intervention and the control group post-intervention, there was a statistically non-significant difference of −5.6% (95% CI, −34.4, 35.5%, *p* = 0.75). The PP analysis for the secondary outcome also revealed a non-statistically significant result (*p* = 0.63) (Table 1).

More than three quarters of intervention GPs (76.0%) were situated in Rotterdam. Therefore, we performed an analysis for this specific subgroup. Descriptive results are shown in the Supplementary Table S2. A non-significant difference of −4.7% in primary outcome was found (95% CI, −32.9, 35.2%, *p* = 0.78) (Supplementary Table S3) and for the secondary outcome there was a non-significant difference of −16.2% (95% CI, −42.7, 22.2%, *p* = 0.35) (Supplementary Table S3). The PP analysis, neither revealed significant results.

### 4.1 (Cross) contamination

Three months after the intervention we asked GPs about possible external influences, other than the PARCA-intervention, that could have affected their antibiotic prescribing behavior. Almost all GPs underlined the influence of the COVID-19 pandemic. GPs also noticed a decrease in the requests of patients for antibiotics.

### 4.2 Self-assessment questionnaire among GPs

In total, 32 GPs filled out the pre- and post-questionnaires. The changes in self-rating on various knowledge and (communication) skills items are presented in Tables 2, 3. There was a statistically significant improvement on four items: ‘*How do you rate your ability to communicate in a culturally-sensitive way with immigrant patients?’* (*p* = 0.005), ‘*How much do you know about people with low health literacy?’* (*p* < 0.001), *‘Do you feel capable to provide adequate care to patients with low health literacy?’* (*p* < 0.001), and ‘*Do you make use of the teach-back method?’* (*p* < 0.001). None of the items that focused on improved knowledge and skills related to antibiotic prescribing were significant. GPs rated the usefulness of the training with a score of 8.3 (range 6–10) right after the intervention and with a score of 7.3 (range 6–9) three months later. Regarding the patient materials, GPs most often used the two texts that are available on the website of the Dutch College of General Practitioners. There were 22 GPs (73.0%) who used these texts regularly or often.

**Table 2:** Self-rating of GPs about their own knowledge and skills pre- and post-intervention (*N* = 32).

	Pre-intervention	Post intervention	Difference pre- and post	Value of *p*
	Mean (SD)	Mean (SD)		
*1. How do you rate your ability to communicate in a cultural-sensitive way with immigrant patients?* (1 absolutely not – 10 excellent)	6.5 (1.07)	7.2 (0.75)	0.69	0.005*
*2. According to your opinion, to which extent do immigrant patients expect to receive antibiotics during a consult?* (1 absolutely not – 10 completely)	7.0 (1.05)	6.7 (1.37)	−0.26#	0.397
*3. How difficult are situations for you in which you do not want to prescribe antibiotics to immigrant patients?* (1 not difficult at all – 10 very difficult)	6.2 (1.67)	5.6 (1.93)	−0.51#	0.084
*4. Do you believe you more often prescribe antibiotics inappropriately for immigrant patients than for native Dutch patients?* (1 absolutely not – 10 always)	5.1 (2.16)	4.5 (2.01)	−0.57#	0.083
*5. Do you believe that immigrant patients understand your arguments for not prescribing antibiotics?* (1 absolutely not – 10 always)	5.9 (1.21)	6.3 (1.31)	0.46	0.142
*6. How much do you know about people with low health literacy?* (1 absolutely nothing – 10 everything)	5.9 (0.98)	6.8 (1.15)	0.91	<0.001*
*7. How do you rate your ability for recognizing patients with low health literacy?* (1 not capable – 10 fully capable)	6.2 (1.26)	6.5 (1.48)	0.25	0.325
*8. Do you feel capable to provide adequate care to patients with low health literacy?* (1 not capable – 10 fully capable)	6.0 (1.25)	6.9 (0.85)	0.97	<0.001*
*9. Do you make use of the teach-back method?* (1 never – 10 always)	5.0 (1.94)	7.0 (1.62)	2.03	<0.001*

^#^A decrease in this item indicates an improvement in knowledge and skills, *p < 0.05. **Wilcoxon Signed rank-test.

**Table 3:** The use and perceived relevance of the developed patient information materials, 3 months after the intervention.

	Answer categories		
How often did you use the following patient materials during your consults? (*N* = 30)	Never	Sometimes	Regularly
*The information texts about antibiotics on Thuisarts.nl*	8 (27%)	19 (63%)	3 (10%)
*(One of) the translations of the texts about antibiotics on Pharos.nl*	16 (53%)	12 (40%)	2 (6%)
*The Dutch animation about antibiotics on Pharos.nl*	20 (67%)	9 (30%)	1 (3%)
*The animation with (one of the) voice-overs in another language than Dutch on Pharos.nl*	19 (63%)	10 (33%)	1 (3%)
To which extend do you agree with the following statements?	Disagree	Neutral	Agree
*The texts about antibiotics provide sufficient support in giving explanation about antibiotics (N = 25)*	2 (8%)	9 (36%)	14 (56%)
*The translations of the texts about antibiotics provide sufficient support in giving explanation about antibiotics (N = 21)*	1 (5%)	7 (33%)	13 (62%)
*The animation movie provides the patient understandable information about antibiotics (N = 18)*	-	6 (33%)	12 (67%)

## 5 Discussion

We aimed to improve antibiotic prescription by enhancing GPs’ communication skills with immigrant patient groups through a communication training and patient materials in multiple languages. The effect evaluation showed no effect of the intervention on the follow-up number of prescribed antibiotics. Yet, there was some improvement in the self-rated knowledge and skills of GPs after participating in the intervention and they rated the usefulness of the intervention for daily practice with a score of 8.3 right after the intervention and with a score of 7.3 three months later.

It can be questioned whether our intervention – which contained adequate power to detect statistical significance – was not effective in changing antibiotic prescribing behavior, or whether we were unable to demonstrate an effect due to the substantial impact of the COVID-19 pandemic. The pandemic affected GPs’ workload, diagnostic possibilities, and the organization of primary care (27, 28), and thereby complicated the recruitment of GPs for our study. The pandemic also directly reduced the incidence of respiratory illness (29) and the number of prescribed antibiotics (24, 30), which explains the decreases in prescribed antibiotics of the control GPs. According to the GPs in our study, a positive impact of the COVID-19 pandemic was the expanded information provision by the government and healthcare organizations. This resulted in better awareness among patients about differences between bacteria and viruses and might have reduced difficult interactions.

The value of effective GP-patient communication to manage patients and to increase mutual understanding is widely emphasized (10, 12, 31). Communication skills training for GPs has been previously proven effective in stimulating more appropriate antibiotic prescribing (10, 15, 32, 33). When training is offered in small groups (34) and includes content about real-life situations, as was done in our PARCA intervention, it usually endorses effective learning and aids in setting learning goals that can be applied in daily practice (35, 36). The results of our self-assessment questionnaire demonstrated an improvement on four knowledge and skills related items. However, none of these items focused on antibiotic prescribing. The items that were related specifically to antibiotic prescribing, for instance *‘Do you believe that immigrant patients understand your arguments for not prescribing antibiotics?’*, did not show any significant improvement. This seems to indicate our intervention was mainly effective in improving GPs’ general communication skills and knowledge, without conjointly influencing their antibiotic prescribing behavior. The use of the teach-back method showed the largest improvement, a method which was intensively practiced during the PARCA intervention. The teach-back method has already been used widely in the community setting and has positive effects on patient outcomes, such as patient satisfaction, perceptions, and disease self-management (37).The other two training elements of our intervention, an online E-learning course, and patient materials, have potential to add to the training (16, 38–40).

A point of criticism of the GPs was that our intervention did not provide a solution for the limited consultation time. Time constraint is repeatedly mentioned as barrier for appropriate antibiotic prescribing (10, 11). On the long term, effective communication can save time as it will aid in a trust-based relationship between the GP and patient (41). To improve one’s own communication skills, an ongoing time investment in practicing and training is required. Continuous education, regular exposure and experiences in real-life situations are needed to develop expertise in the communication with vulnerable patients (42).

Recent studies have shown that most immigrants have similar attitudes and expectations as the general population (41) and that they have adapted their antibiotic attitude to the host country (43). Still, communication between GPs and immigrant patients requires constant attention. Information from healthcare professionals to patients is often inadequate and, despite our globalizing world, support from written information in the migrants’ mother tongue language is scarce. Moreover, currently used translation methods, such as informal translators, are not always sufficient (41, 42, 44).

The effect evaluation of our study focused on quantitative outcome measures. For future research it is recommended to use other (qualitative) methods like video observations or interviews, that provide the opportunity to measure the influence of the intervention on communication skills of GPs and possibly patients’ reassurance, satisfaction and understanding. Subsequently, while there is support for the influence of effective communication skills (15, 45), the use of other methods could provide in-depth knowledge about the added value of learning culturally-sensitive communication skills.

### 5.1 Strengths and limitations

Our study provides a valuable contribution to primary care practice because it is one of the first studies that has focused on immigrant patient groups to improve antibiotic prescribing. Furthermore, the PARCA intervention received a high rating from participating GPs, resulted in improved knowledge and skills, and we noticed a broad interest in our communication training during the intervention. By offering the live training as an in-company training, other interested employees, who were legally allowed to prescribe medications (e.g., nursing specialists), used the opportunity to also participate.

An important limitation of our study was the impossibility to randomize the participating GPs. Because of low willingness to participate in our study, even after extending our recruitment to GPs working in other (non-deprived) areas, the performance of a randomized controlled trial (RCT), as originally intended (25), was not achievable. Consequently, we included all recruited GPs in the intervention group and compared their prescription data with an anonymous control group, using a before-after study design. But even though this design ranks lower on hierarchy of evidence (46, 47), we believe it provides valuable insights. The results demonstrated that there were no differences between intervention and control GPs post-intervention when adjusting for the pre-intervention number of prescribed antibiotics, and there is no reason to expect another outcome if we had performed an RCT. Yet, there might be an underestimation of the effect as we can expect that particularly GPs with interest in the subject participated in the training, while they probably not perform the worst regarding antibiotic prescribing. In line, several GPs mentioned to have participated in various antibiotic oriented and/or communication courses, and our data revealed that the number of prescribed antibiotics was consistently lower among the intervention GPs than among the control GPs.

Another limitation of the study, that results from including anonymous GPs in the control group, is that we could not collect data about the specific number of registered patients for each GP who participated. This forced us to change the primary outcome measure that we originally intended to use, the number of antibiotic courses qualifying for RTI per 1,000 registered patients, to the absolute number of antibiotic courses qualifying for RTI. Similarly, the secondary outcome measure was changed to the absolute number of all prescribed antibiotic courses instead of per 1,000 registered patients. As a consequence, we also needed to change our sample size calculation. But, because the alternative design enabled us to include a larger number of GPs in the control group, we could increase the power of our study. Also, the anonymous GPs in the control group hampered us to statistically adjust for possible differences between intervention and control GPS, as no data on background characteristics such as work experience, age or type of practice were available for these anonymous GPs. This may also explain the initial difference in prescribed antibiotics between intervention and control GPs, in that control GPs might have more patients and/or work more hours.

Another limitation is related to the lack of information about any patient characteristics. Due to data restrictions it was impossible to select immigrant patients with symptoms of an RTI for our outcome measures. Data that had included only immigrant patients, instead of all patients as in our current data file, would have been more appropriate as our intervention was focused specifically on improving GPs’ communication with immigrant patients. It could have been possible that the share of immigrant patients in the practices of some of the intervention GPs was too small to demonstrate any effect. Finally, regarding antibiotic prescription for patients with RTI, we used a selection of antibiotics that qualify for RTIs as a proxy for antibiotics that can be prescribed when a patient is diagnosed with an RTI. However, these antibiotics can also be prescribed for other infections. Ideally, we would have liked to extract all consultations for RTI from GPs’ medical files and calculated prescribing rates, which was not possible given the various systems that were in place, as well as budget and time constraints.

The absence of an effect of the PARCA intervention on antibiotic prescribing by GPs might indicate that the intervention was ineffective but could also mean that the collected data and timing of the intervention were suboptimal. Further research is needed to examine the effect of improved culturally-sensitive versus effective communication skills on the prescribing behavior of GPs and patients’ satisfaction, by using a mix of both quantitative and qualitative methods.

## Data availability statement

The generated datasets for this study will become available via the website: www.zorggegevens.nl of the National Institute for Public Health and the Environment. The data can be required upon request. Requests to access these datasets should be directed to Petra de Vries, p.devries.3@erasmusmc.nl.

## Ethics statement

Ethical approval for this study was waived by the Medical Ethics Review Committee of the Erasmus MC, University Medical Center Rotterdam (MEC-2020-0142) since the intervention targeted GPs and we did not analyze or include patients’ health outcomes.

## Author contributions

DL: Conceptualization, Formal analysis, Investigation, Methodology, Project administration, Writing – original draft. ÖE: Formal analysis, Methodology, Writing – review & editing. DN: Formal analysis, Methodology, Writing – review & editing. NH: Conceptualization, Funding acquisition, Investigation, Project administration, Writing – review & editing. AT-A-T: Conceptualization, Writing – review & editing. WO: Conceptualization, Writing – review & editing. RB: Conceptualization, Writing – review & editing. MV: Writing – review & editing. AV: Conceptualization, Methodology, Writing – review & editing. JR: Conceptualization, Funding acquisition, Investigation, Methodology, Project administration, Supervision, Writing – review & editing. HV: Conceptualization, Funding acquisition, Investigation, Methodology, Project administration, Supervision, Writing – review & editing.

## Footnotes

^1^ www.sfk.nl/english
