# Supplementary material for: Communication training for general practitioners aimed at improving antibiotic prescribing: a controlled before-after study in multicultural Dutch cities
Source: Front Med (Lausanne). 2024 Jan 23;11:1279704. doi: 10.3389/fmed.2024.1279704 (PMC10844435; doi:10.3389/fmed.2024.1279704)
Supplement: Supplementary file 1 [file Table_1.docx]

Supplementary Material

Communication training for general practitioners aimed at improving antibiotic prescribing: a controlled before-after study in multicultural Dutch cities

Dominique L.A. Lescure^*^, Özcan Erdem, Daan Nieboer, Natascha Huijser van Reenen, Aimée M.L. Tjon-A-Tsien, Wilbert van Oorschot, Rob Brouwer, Margreet C. Vos, Alike W. van der Velden, Jan Hendrik Richardus, Hélène A.C.M. Voeten

*** Correspondence:** [dla.lescure@rotterdam.nl](mailto:dla.lescure@rotterdam.nl)

# Supplementary Tables

Table S1: Characteristics of the intervention GPs (N=25).

|  | *N=25* | *%* |
| --- | --- | --- |
| *Sex* |  |  |
| Men | 6 | 24% |
| Woman | 19 | 76% |
|  |  |  |
| *Age* |  |  |
| 30-40 | 7 | 28% |
| 41-50 | 4 | 16% |
| 51-60 | 7 | 28% |
| >60 | 2 | 8% |
| Unknown | 5 | 20% |
|  |  |  |
| *Proportion of FTE* |  |  |
| ≤0.5 | 3 | 12% |
| 0.6-0.7 | 8 | 32% |
| 0.8-0.9 | 3 | 12% |
| >0.9 | 6 | 24% |
| Unknown | 5 | 20% |
|  |  |  |
| *Years of work experience* |  |  |
| <5 years | 4 | 16% |
| 5-10 years | 5 | 20% |
| ≥10 years | 11 | 44% |
| Unknown | 5 | 20% |
|  |  |  |
| *City* |  |  |
| Rotterdam | 19 | 76% |
| Amsterdam | 4 | 16% |
| The Hague | 1 | 4% |
| Other | 1 | 4% |
|  |  |  |
| *Participated in the intervention (live training and/or E-learning)* | 19 | 76% |
|  |  |  |
| *Working in a deprived area* | 19 | 76% |
