# Supplementary material for: Communication training for general practitioners aimed at improving antibiotic prescribing: a controlled before-after study in multicultural Dutch cities
Source: Front Med (Lausanne). 2024 Jan 23;11:1279704. doi: 10.3389/fmed.2024.1279704 (PMC10844435; doi:10.3389/fmed.2024.1279704)
Supplement: Supplementary file 2 [file Table_2.docx]

Supplementary Material

Communication training for general practitioners aimed at improving antibiotic prescribing: a controlled before-after study in multicultural Dutch cities

Dominique L.A. Lescure^*^, Özcan Erdem, Daan Nieboer, Natascha Huijser van Reenen, Aimée M.L. Tjon-A-Tsien, Wilbert van Oorschot, Rob Brouwer, Margreet C. Vos, Alike W. van der Velden, Jan Hendrik Richardus, Hélène A.C.M. Voeten

*** Correspondence:** [dla.lescure@rotterdam.nl](mailto:dla.lescure@rotterdam.nl)

Table S2: Number of prescribed antibiotics per GP, RTI-related and overall, for the intervention group (*N*=19) and control group (*N*=110), pre-intervention (2019-2020) and post-intervention (2021-2022) - a selection of GPs from Rotterdam

|  | **Intervention GPs (N=19)** | | **Reference GPs (N=37)** | |
| --- | --- | --- | --- | --- |
|  | Baseline 2019 - 2020 | Follow-up 2021 – 2022 | Baseline  2019 - 2020 | Follow-up  2021 - 2022 |
| **Antibiotics for RTI** |  |  |  |  |
| Absolute number | 2930 | 2514 | 9692 | 9677 |
| Mean (SD) | 154 (91,12) | 132 (98,41) | 261 (292,07) | 262 (334,93) |
| Median of prescribed antibiotics | 151 | 112 | 130 | 109 |
| Range of prescribed antibiotics | 48 – 406 | 18 – 451 | 27 – 1506 | 15 – 1681 |
|  |  |  |  |  |
| **Total number of antibiotics** |  |  |  |  |
| Absolute number | 4569 | 4192 | 13603 | 14074 |
| Mean (SD) | 240 (135,10) | 221 (173,30) | 368 (362,07) | 380 (402,27) |
| Median of prescribed antibiotics | 221 | 190 | 199 | 204 |
| Range of prescribed antibiotics | 75 – 630 | 27 – 833 | 12 – 1782 | 22 – 1935 |
