# Supplementary material for: Communication training for general practitioners aimed at improving antibiotic prescribing: a controlled before-after study in multicultural Dutch cities
Source: Front Med (Lausanne). 2024 Jan 23;11:1279704. doi: 10.3389/fmed.2024.1279704 (PMC10844435; doi:10.3389/fmed.2024.1279704)
Supplement: Supplementary file 3 [file Table_3.docx]

Supplementary Material

Communication training for general practitioners aimed at improving antibiotic prescribing: a controlled before-after study in multicultural Dutch cities

Dominique L.A. Lescure^*^, Özcan Erdem, Daan Nieboer, Natascha Huijser van Reenen, Aimée M.L. Tjon-A-Tsien, Wilbert van Oorschot, Rob Brouwer, Margreet C. Vos, Alike W. van der Velden, Jan Hendrik Richardus, Hélène A.C.M. Voeten

*** Correspondence:** [dla.lescure@rotterdam.nl](mailto:dla.lescure@rotterdam.nl)

Table S3: ANCOVA analysis (intention to treat (ITT) and per protocol (PP)) of the mean number of prescribed antibiotics for RTI and the mean number of prescribed antibiotics for all infections in the intervention group (subgroup of Rotterdam GPs ITT, *N*=19; subgroup of Rotterdam GPs PP, *N*=14) compared to the reference group (*N*=37), post-intervention, unadjusted and adjusted for the baseline number of prescribed antibiotics*

|  | **Post-test** | |  | **Adjusted post-test** | |  |
| --- | --- | --- | --- | --- | --- | --- |
|  | Intervention group  (per GP) | Reference group  (per GP) | Difference in the mean number of prescribed antibiotics (intervention vs. reference group) | Intervention group  (per GP) | Reference group  (per GP) | Difference in the mean number of prescribed antibiotics (intervention vs. reference group) |
| ***Intention to treat (ITT)*** |  |  |  |  |  |  |
| Mean number of prescribed AB for RTI | 104 | 134 | 22.2% | 119 | 125 | 4.7% |
| 95% CI | 64 – 170 | 94 – 190 | -57.2% – 41.9% | 90 – 158 | 102 – 153 | -32.9% – 35.2% |
| *p* |  |  | 0.407 |  |  | 0.782 |
|  |  |  |  |  |  |  |
| Mean number of total prescribed AB | 175 | 225 | 22.4% | 184 | 220 | 16.2% |
| 95% CI | 111 – 275 | 163 – 312 | -55.5 – 35.5% | 135 – 250 | 176 – 274 | -42.7% – 22.2% |
| *p* |  |  | 0.366 |  |  | 0.348 |
|  |  |  |  |  |  |  |
| ***Per protocol (PP)*** |  |  |  |  |  |  |
| Mean number of prescribed AB for RTI | 88 | 134 | 34.7% | 104 | 125 | 16.8% |
| 95% CI | 49 – 157 | 94 – 192 | -67.0% – 29.4% | 75 – 145 | 103 – 153 | -43.5% – 22.5% |
| *p* |  |  | 0.217 |  |  | 0.344 |
|  |  |  |  |  |  |  |
| Mean number of total prescribed AB | 146 | 225 | 35.3% | 160 | 217 | 26.4% |
| 95% CI | 85 – 249 | 162 – 313 | -65.5% – 21.6% | 174 – 272 | 174 – 272 | -51.9% – 13.1% |
| *p* |  |  | 0.172 |  |  | 0.156 |

* The data were transformed by using LOG10 transformation and back-transformed by using the logarithmic operation in reverse.
